# Cardiac Rhythm Device Identification Using Neural Networks

**DOI:** 10.1016/j.jacep.2019.02.003

**Published:** 2019-05

**Authors:** James P. Howard, Louis Fisher, Matthew J. Shun-Shin, Daniel Keene, Ahran D. Arnold, Yousif Ahmad, Christopher M. Cook, James C. Moon, Charlotte H. Manisty, Zach I. Whinnett, Graham D. Cole, Daniel Rueckert, Darrel P. Francis

**Affiliations:** a Department of Cardiology, National Heart and Lung Institute, Imperial College London, London, United Kingdom; b Department of Cardiology, University College London, London, United Kingdom; c Department of Computing, Imperial College London; London, United Kingdom

**Keywords:** cardiac rhythm devices, machine learning, neural networks, pacemaker, AP, anterior-posterior, ICD, implantable cardioverter- defibrillator, PA, posterior-anterior

## Abstract

**Objectives:**

This paper reports the development, validation, and public availability of a new neural network-based system which attempts to identify the manufacturer and even the model group of a pacemaker or defibrillator from a chest radiograph.

**Background:**

Medical staff often need to determine the model of a pacemaker or defibrillator (cardiac rhythm device) quickly and accurately. Current approaches involve comparing a device’s radiographic appearance with a manual flow chart.

**Methods:**

In this study, radiographic images of 1,676 devices, comprising 45 models from 5 manufacturers were extracted. A convolutional neural network was developed to classify the images, using a training set of 1,451 images. The testing set contained an additional 225 images consisting of 5 examples of each model. The network’s ability to identify the manufacturer of a device was compared with that of cardiologists, using a published flowchart.

**Results:**

The neural network was 99.6% (95% confidence interval [CI]: 97.5% to 100.0%) accurate in identifying the manufacturer of a device from a radiograph and 96.4% (95% CI: 93.1% to 98.5%) accurate in identifying the model group. Among 5 cardiologists who used the flowchart, median identification of manufacturer accuracy was 72.0% (range 62.2% to 88.9%), and model group identification was not possible. The network’s ability to identify the manufacturer of the devices was significantly superior to that of all the cardiologists (p < 0.0001 compared with the median human identification; p < 0.0001 compared with the best human identification).

**Conclusions:**

A neural network can accurately identify the manufacturer and even model group of a cardiac rhythm device from a radiograph and exceeds human performance. This system may speed up the diagnosis and treatment of patients with cardiac rhythm devices, and it is publicly accessible online.

More than 1 million people worldwide undergo implantation of a cardiac rhythm device every year (1), which includes pacemakers, defibrillators, and loop recorders. This number continues to grow (2) as indications increase and modern health care becomes more accessible to more people. Specialized staff can use specific communication equipment to interrogate and program these devices, but to do this they need to know the device’s manufacturer, so they can bring the corresponding communication equipment to the bedside.

Unless they have access to the records of the implanting hospital or the patient can tell them, staff must use a process of trial and error to identify the manufacturer, which causes uncertainty and leads to delays which can be medically harmful.

Experts can sometimes distinguish among devices from a chest radiograph, and algorithms are available to assist with this. However, expertise or confidence in using the algorithm are not widespread, and even with the best available algorithm, identification is not perfect. Indeed, up to 80% of physicians report having “frequently” had difficulties identifying devices (3).

The most recent algorithm for visual discrimination among devices shown on a chest radiograph is 8 years of age (3) and therefore does not include current devices. Even at that time, the study authors reported only 90% accuracy in identifying the manufacturer.

The present study reports the development, validation, and public availability of a new neural network-based system which attempts to identify the manufacturer and even the model group of a device by using a chest radiograph.

## Methods

### Data extraction

In this study, a dataset was constructed of radiographic images of devices implanted in adults at Imperial College Healthcare NHS Trust between February 1998 and May 2018. Training a neural network requires an adequate number of examples of each class to be identified; therefore, only device models were included for which there were at least 25 chest radiographic images available. Both portable and departmental anterior-posterior/posterior-anterior (AP/PA) chest radiographs were included. Lateral chest radiographs were not included. In the absence of any data outlining the prevalence of different devices across the world, a dataset was generated in which all types of device were represented in equal proportions. Images were extracted from consecutive patients to a maximum of 40 images per model to minimize class imbalance (4). From each radiographic image, a square region of interest slightly larger than the device was extracted. This region maximized the signal-to-noise ratio for the network and guaranteed anonymization. These cropped images were then resized to 224 × 224 pixels and normalized to yield pixel values between 0 and 1. It was noted during extraction that, in several cases, when a manufacturer introduced a new model, there was no detectible change on the radiograph. This may represent purely a change in software or an indistinguishable replacements of parts; therefore, models with identical appearance were placed in “model groups.”

The first step was to randomly allocate 5 images from each of the 45 classes to be kept aside as the final “test set.” This would not be shown to the network at any stage in its training and would only be used once when reporting its final accuracy.

The remaining “training set” was used to train the network at 2 different stages. The first stage was to decide which underlying network to use (including structural features such as the number and size of layers) and details of how the training process would run (including the avidity with which synapses are adjusted, termed the “learning rate”). All tested neural networks were convolutional neural networks which contain neurons that learn to recognize specific features within their own “visual fields.” These networks are organized in a hierarchical structure akin to the human optic cortex and excel at solving image classification problems 5, 6, 7, 8, 9. The second stage was the detailed process of adjusting the weights (akin to the synapses in a biological neural network) so that the job of classifying pacemakers could be performed. Both stages used the training set but in different ways.

**Figure 1:**
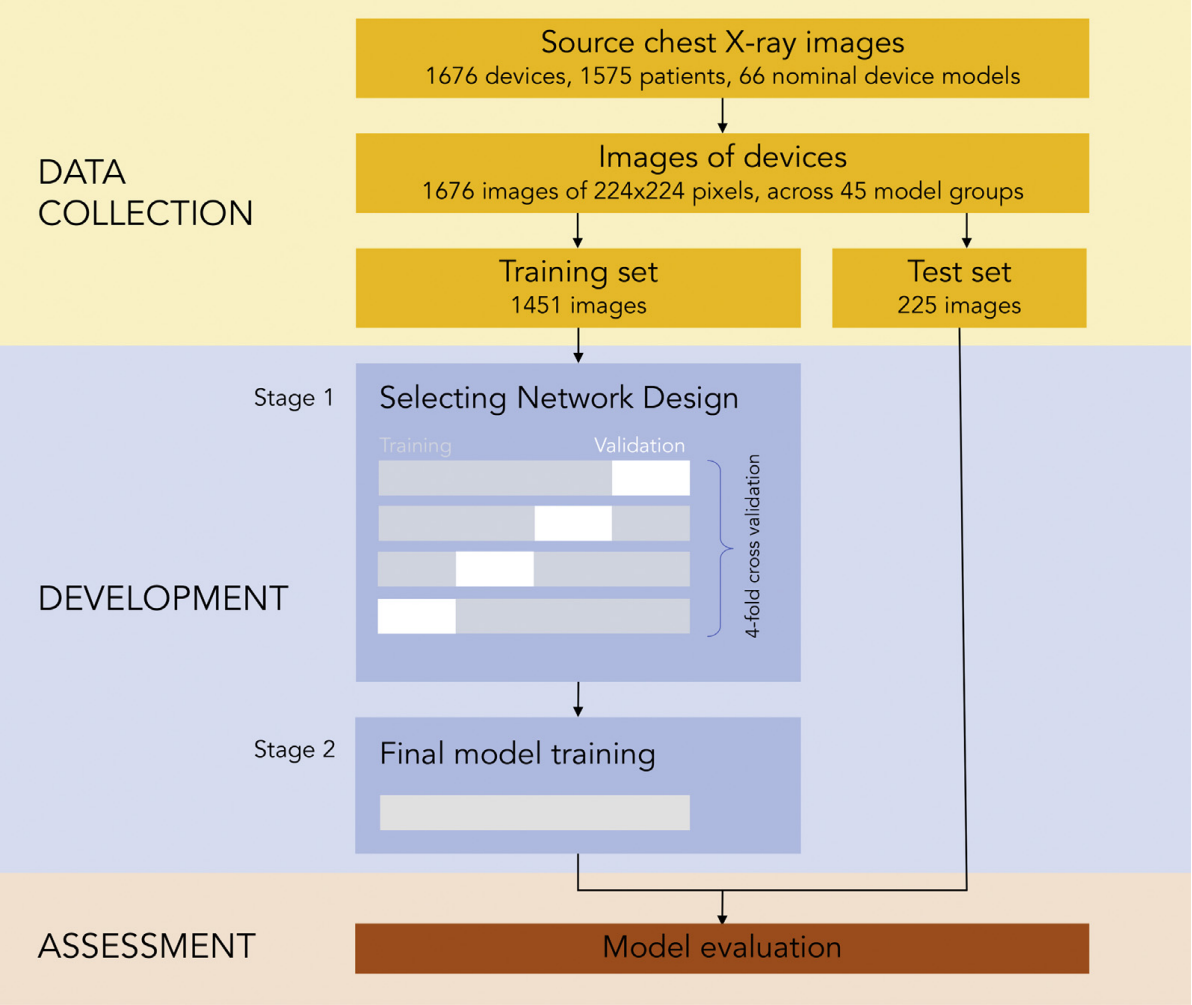
Study Design Flowchart The study was designed in 3 phases consisting of data collection, development of the neural network, and assessment of the network. Development of the neural network was divided into 2 stages. Stage 1 involved selecting the optimal network design. Stage 2 involved training the “final” model, which is then assessed using the unseen “test set”, allowing a comparison with humans.

For the first stage (“network design”) (Figure 1), each candidate neural network design was assessed by its ability to learn from 75% of the training set and correctly make predictions for the remaining 25% of the training set. This was done 4 times, so that all of the training set could participate in turn in both roles. This process is termed “4-fold cross-validation” (unrelated to the final testing which is performed using a completely separate test set).

The second stage (“final model training”) begins with the neural network design chosen by the first stage. This starts with a fresh neural network with no prior exposure to device images. The network is then trained from the entire training set, resulting in the final trained network.

Finally, this final network is exposed for the first time to the “test set,” which has been kept separate throughout. It is assessed for its ability to correctly classify manufacturer and model group.

Regulatory approval for the study was granted by the Health Research Authority (Integrated Research Application System identifier 249461).

### Convolutional neural network architecture and training

Five different convolutional neural network architectures were assessed (DenseNet, Inception V3, VGGNet, ResNet and Xception), all of which have at some stage in recent years been the world leading design for the ImageNet dataset challenge 5, 6, 7, 8, 9. All networks were initialized using weights derived from training on ImageNet before the whole model was retrained. Regularization and dropout were implemented for each network according to their original publications.

For each network, output layer was set to have 45 densely connected neurons (1 for each device model group). Loss was calculated over batches of 16 images by using the categorical cross-entropy loss function, and weights were updated using the ADADELTA optimizer (10). Loss is a technical marker used to assess the network’s performance and make improvements. Loss is more sensitive than simply the misclassification rate (the inverse of accuracy), because to achieve full marks (zero loss), the network has, for each pacemaker image, to be 100% confident in the correct prediction and have 0% suspicion for all 44 other model groups. Training of the neural network is an automatic process of adjusting the synapse weights to minimize this loss. Training continued until validation loss plateaued (15 epochs). Training was augmented with random rotation, width and height shifting, vertical and horizontal flipping, and shearing and zooming. Programming was performed using the Python programming language with the Tensorflow (11) and Keras (12) machine learning frameworks. Training was performed on 2 GeForce GTX 1080 Ti graphical processing units (nVIDIA, Santa Clara, California).

### Visualization of learning

Examples of each model group were processed to provide saliency maps (13) where the pixels with the highest gradient with regard to the correct class (i.e., the pixels contributing most to the decision of the network) were highlighted. This was performed using Keras-vis software (14).

### Human expert performance using manual algorithm

The test sets of 225 images was supplied to 5 independent cardiologists (2 of whom were electrophysiologists) along with the full manuscript of the Cardiac Rhythm Device Identification Algorithm Using X-rays (CaRDIA-X) algorithm, the most recent algorithm for classifying cardiac devices from chest radiographs (3). The algorithm only aims to distinguish among manufacturers rather than to identify the particular model group. With each image, graders were informed as to whether the device was a pacemaker, a defibrillator, or a loop recorder. Graders were asked to classify each device as Biotronik (Lake Oswego, Oregon), Boston Scientific (including Guidant and Cameron Health, Marlborough, Massachusetts), Medtronic (including Vitatron, Fridley, Minnesota), Sorin (including Liva Nova, Arvada, Colorado) or St. Jude Medical (Little Canada, Minnesota).

### Statistical analysis

The prespecified primary endpoint was a superiority test result for manufacturer accuracy between the artificial network and human expert graders, using the CaRDIA-X manual algorithm. Results were assessed using McNemar’s test with a p value of 0.05 as the threshold for statistical significance, with an exact test used for contingency Tables including any counts below 25. Because each human grader is an individual, the primary endpoint was calculated with reference to the human grader with the median accuracy.

Accuracy was defined as the number of correctly classified images in the test set divided by the total number of images in the test set. Confidence intervals (CIs) for accuracy were calculated using the “exact” binomial method. For manufacturer accuracy where class sizes were inherently unequal, the F_1_ score was also calculated, defined as double the harmonic average of the precision and recall, bounded between 0 and 1.

The accuracy of the network was assessed across several subgroups (departmental vs. portable radiographs, pacemakers vs. implantable cardioverter-defibrillator [ICDs], and across the different device manufacturers) by using the Fisher's exact test. Welch’s unequal variances *t*-test was used to assess for differences in image quality (sharpness) between departmental and portable radiographs by calculating the variance of Laplacian for each image (15). Statistical analysis was performed using R software (R Foundation, Vienna, Austria) (16).

## Results

### Dataset

The full dataset consisted of 1,676 images of unique devices from 1,575 unique patients (some patients had more than 1 device during the study period). Although there were 66 different nominal device models, several of them were visually indistinguishable from each other, perhaps representing purely software changes between models. There were 45 different model groups with unique radiographic appearances (Table 1). A total of 278 radiographs (16.6%) were from portable radiography machines; the remaining 1,398 devices (83.4%) were departmental AP or PA radiographs. Online Appendix 1 shows each model group and the nominally different models with identical appearances that comprised that model group.

**Table 1:** Distribution of Classes Across the Entire Dataset

Manufacturer	Nominal Model	n	Model Group	n
Pacemaker (n = 1,213)				
Biotronik	Actros	20	Actros/Philos	40
	Philos	20		
	Cyclos	27	Cyclos	27
	Evia	28	Evia	28
Boston Scientific	Altrua	20	Altrua/Insignia	40
	Insignia	20		
	Contak Renewal TR2	40	Contak Renewal TR2	40
	Contak TR	10	Contak TR/Discovery/Meridian/Pulsar Max	40
	Discovery	10		
	Meridian	10		
	Pulsar Max	10		
	Ingenio	40	Ingenio	40
	Proponent	40	Proponent	40
	Visionist	40	Visionist	40
Medtronic	Adapta	10	Adapta/Kappa/Sensia/Versa	40
	Kappa	10		
	Sensia	10		
	Versa	10		
	Advisa	40	Advisa	40
	AT500	38	AT500	38
	Azure	40	Azure	40
	C20	20	C20/T20	40
	T20	20		
	C60	40	C60	40
	Enrhythm	40	Enrhythm	40
	Insync III	40	Insync III	40
	Sigma	40	Sigma	40
	Syncra	40	Syncra	40
	Vita II	29	Vita II	29
Sorin	Elect	40	Elect	40
	Elect XS Plus	30	Elect XS Plus	30
	MiniSwing	28	MiniSwing	28
	Neway	37	Neway	37
	Reply	40	Reply	40
	Rhapsody	20	Rhapsody/Symphony	40
	Symphony	20		
	Thesis	36	Thesis	36
St. Jude	Accent	40	Accent	40
	Allure Quadra	40	Allure Quadra	40
	Identity	40	Identity	40
	Victory	40	Victory	40
	Zephyr	40	Zephyr	40
ICD (n = 415)				
Boston Scientific	Autogen	10	Autogen/Cognis/Energen/Teligen	40
	Cognis	10		
	Energen	10		
	Teligen	10		
	Contak Renewal 4	40	Contak Renewal 4	40
	Emblem	40	Emblem	40
	Ventak Prizm	33	Ventak Prizm	40
	Vitality	40	Vitality	40
Medtronic	Claria	13	Claria/Evera/Viva	40
	Evera	13		
	Viva	14		
	Concerto	8	Concerto/Consulta/Maximo/Protecta/Secura	40
	Consulta	8		
	Maximo	8		
	Protecta	8		
	Secura	8		
	Maximo	30	Maximo	30
Sorin	Ovatio	25	Ovatio	25
St. Jude	Ellipse	40	Ellipse	40
	Quadra Assura	40	Quadra Assura	40
Loop recorders (n = 58)				58
Medtronic	Reveal	26	Reveal	26
	Reveal Linq	32	Reveal Linq	32

Distribution of classes across the entire dataset is divided into device type, manufacturer, and model. Visually identical model names (middle column) are merged into “model groups” (right column). ICD = implantable cardioverter-defibrillator.

The test set consisted of 5 examples of each of the 45 final model groups to make a total of 225 examples. Thirty-eight radiographs (16.9%) were portable radiographs. The remaining 1,451 cases were assigned to the training set. The study flow chart is shown in Figure 1.

### Stage 1: Comparative performance of the different neural network architectures

For all network designs, after 15 epochs of training in stage 1, the network had reached a plateau of performance, manifesting as a plateau after the initial decline in the validation loss. Table 2 shows the level of this plateau in the validation loss and corresponding validation accuracy for each of the 5 network architectures assessed. Each number displayed is the averaged value over the 4 splits of the training set. The accuracy varied from 4.4% for VGGNet to 91.1% for Xception.

**Table 2:** Distribution of Classes Across the Entire Dataset

Architecture (Ref. #)	Trainable Parameters (millions)	Loss (Lower Is Better)	% of Accuracy (Higher Is Better)
DenseNet 121 (9)	7.0	0.36	90.8
Inception V3 (6)	21.9	1.06	79.5
Resnet (7)	23.6	3.24	44.9
VVGNet 16 (5)	14.7	4.33	4.4
Xception (8)	20.9	0.34	91.1

Results of stage 1, in which the 5 architectures are compared, having been trained on only three-fourths of the training data at a time. Performance of 5 network designs. Loss is a special index of inaccuracy which gives penalties for confident wrong predictions more than unconfident ones.

Based on these results, the conclusion of stage 1 was to select the Xception architecture for stage 2 and to prespecify that the number of epochs of training would be 15. Stage 2, therefore, began with a fresh neural network of the Xception architecture and performed 15 epochs of training using the full training set of 1,451 images.

Finally, the “test set” of data which had been set aside until now was tested, once, using the final neural network produced by stage 2.

### Stage 2: Final neural network performance on the unseen “test set.”

The accuracy of the final neural network for identifying the manufacturer of a device was 99.6% (95% CI: 97.5% to 100.0%), corresponding to an F_1_ score of 0.996. The performance is shown as a confusion matrix in the Central Illustration (right panel). The only image wrongly classified was that of a Medtronic Adapta device which was mistaken for a Sorin Reply device (Online Appendix 2 page 4).

**Central Illustration:**
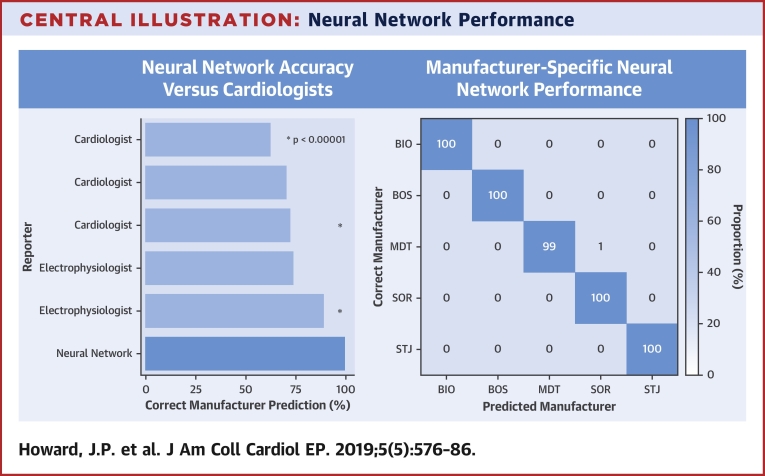
Neural Network Performance **(Left)** Bar plot shows comparative accuracy for identifying the manufacturer of devices across the 5 human reporters and the neural network. The p values are for superiority of the neural network above the median and best human graders. **(Right)** Confusion matrix shows the accuracy of the network in predicting the correct manufacturer of devices. BIO = Biotronik; BOS = Boston Scientific; MDT = Medtronic; SOR = Sorin; STJ = St. Jude.

**Figure 2:**
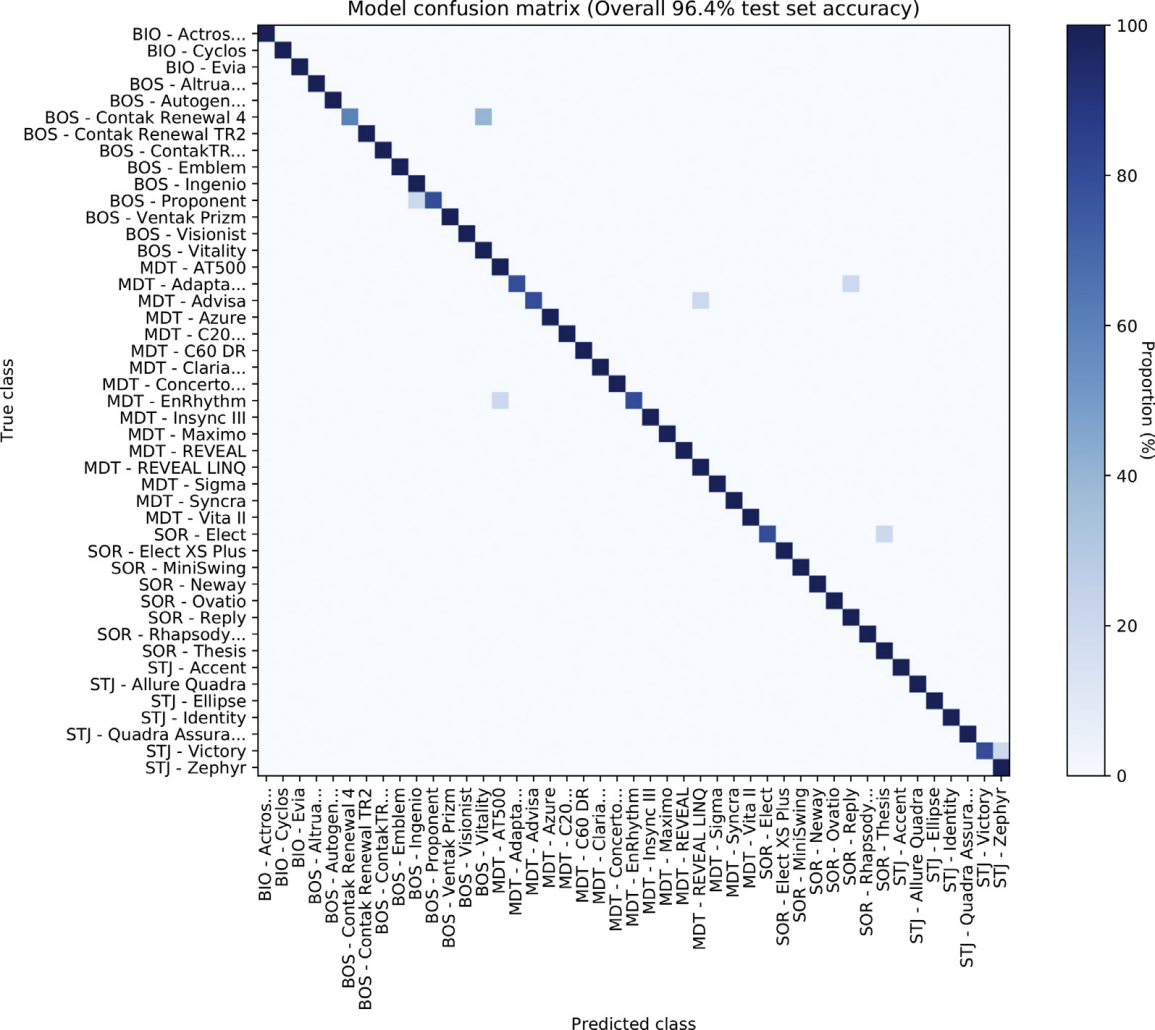
Confusion Matrix For Model Group Identification Confusion matrix shows the accuracy of the network in predicting the correct model of devices. Class names ending in ellipses (“…”) refer to those including more than 1 device type with identical appearance. BIO = Biotronik; BOS = Boston Scientific; MDT = Medtronic; SOR = Sorin; STJ = St. Jude.

Inevitably, performance for identifying the model group (rather than only the manufacturer) was lower. Accuracy was 96.4% (95% CI: 93.1% to 98.5%), and the F_1_ score was 0.964. Figure 2 shows the confusion matrix. The 8 images for which the neural network suggested an incorrect model group are all shown in Online Appendix 2, in each case along with the top 3 predictions for the model group. Notably, in 7 of 8 of these, the correct model group was 1 of the top 3 predictions. Therefore, what is commonly described as the “top 3” accuracy was 99.6% (95% CI: 97.5% to 100.0%) for model group.

The model group accuracy for portable radiographs was 89.5% (95% CI: 75.2% to 97.1%) versus 97.9% (95% CI: 94.6% to 99.4%) for departmental radiographs (p = 0.029 for differences between the 2 groups). This accuracy corresponded with departmental X-ray images being significantly sharper (as judged by the variance of Laplacian) compared to portable X-rays (p < 0.0001 for difference between the two groups). The single manufacturer classification error was of a departmental radiograph, however. Model group accuracy for pacemakers was 95.0% (95% CI: 90.4% to 97.8%) versus 96.4% (95% CI: 87.5% to 99.6%) for ICDs (p = 1.00 for difference between the 2 groups). Model group accuracy did not vary significantly among different manufacturers (p = 0.954).

### Comparison with performance of human experts using CaRDIA-X algorithm

Five human cardiologists applied the published CaRDIA-X algorithm to classify the 225 test set images among the 5 manufacturers. Their accuracy ranged from 62.3% to 88.9%. The median accuracy was 72.0%. The 2 humans who performed the best were the 2 electrophysiologists. The median human performance was that of the best-performing nonelectrophysiologist cardiologist.

The neural network was significantly more accurate than both the median (odds ratio [OR]: 63.0; 95% CI: 10.9 to 2,527.4; p < 0.0001) and the best-performing human grader (OR: 25.0; 95% CI: 4.1 to 1,026.4; p < 0.0001) as shown in the Central Illustration (left panel).

### Visualizing learning with saliency mapping

In an additional exploratory analysis, saliency maps were produced for each image in the test set, indicating the features of each image which were most characteristic of the pacemaker they depicted. These are akin to the pathognomonic signs of a disease in clinical medicine.

**Figure 3:**
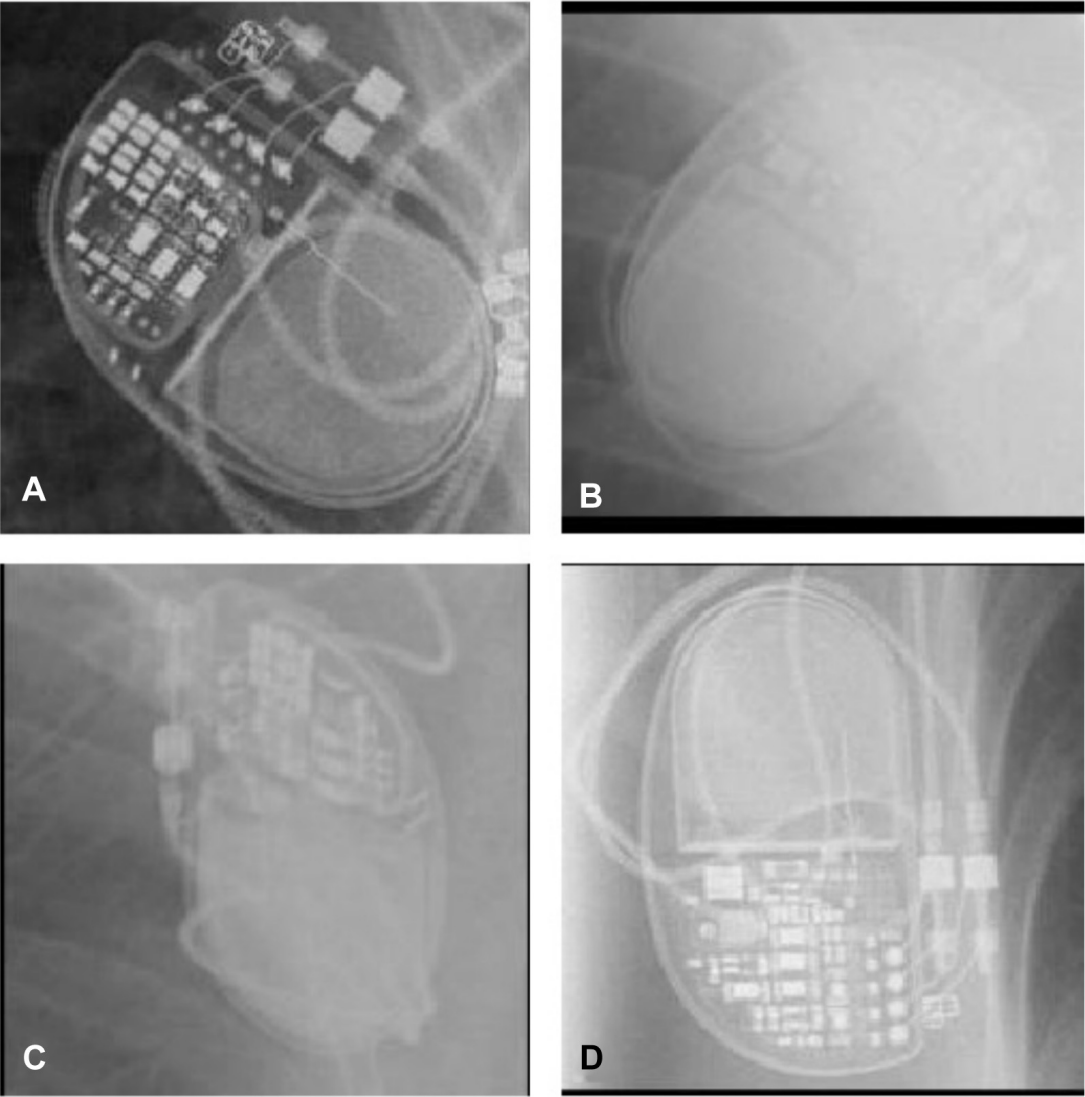
Where to Look? Four images depict 2 Advisa devices and 2 AT500 devices. **(A)** [Shows an] Advisa [model]. Readers are invited to identify which other panel **(B**, **C**, **orD)** is also an Advisa. The other 2 are AT500s. Additionally, how would you advise others to make the same distinction? Once you have made up your mind, consult Figure 4.

**Figure 4:**
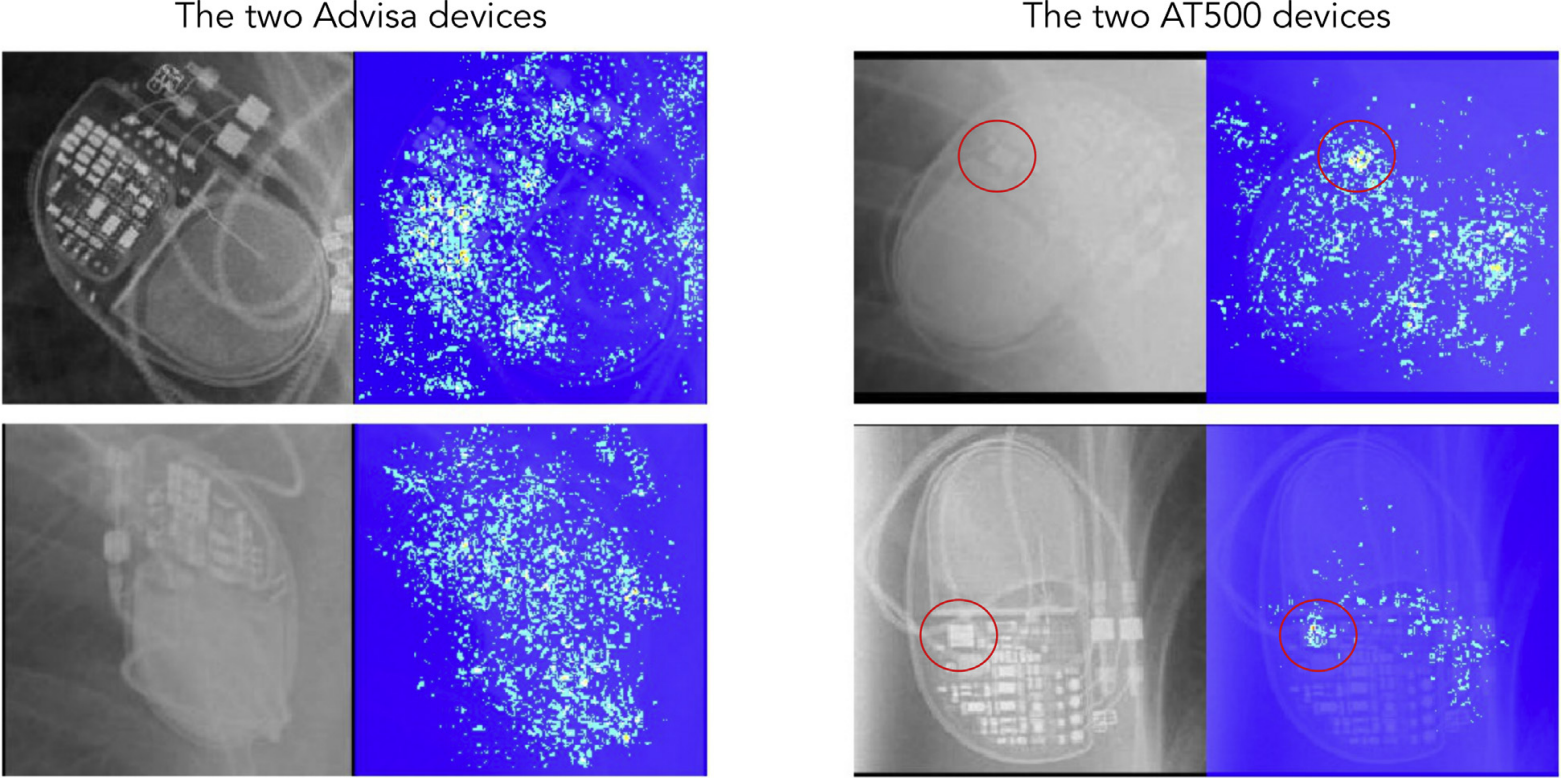
Saliency Plots Saliency plots from the neural network can help guide us where to look. The answer to the question in Figure 2 is **C**. Saliency plots reveal that the network is focusing on a feature present in the AT500s **(red circles)**, which is absent in the Advisas. Having this pointed out by the network now makes it easy to return to Figure 3 and correctly categorize them. These example images also demonstrate the neural network’s ability to deal with dramatic differences in image quality, radiography, penetration, and orientation.

To demonstrate the utility of saliency mapping, Figure 3 shows 4 images comprising 2 different model groups. Figure 3A shows a Medtronic Advisa pacemaker. Readers are invited to identify which other panel (Figure 3B, 3C, or 3D) is also an Advisa and to ask how they would teach others to differentiate between the 2 model groups on a radiograph. Once they have done this, readers are invited to examine Figure 4. This shows that the saliency maps for the AT500 device show intense activity around a circled circuit board component which is unique to that device. Hopefully readers agree that, on revisiting Figure 3 with this knowledge, 2 model groups are easily differentiated. Examples of saliency maps for each of the 45 classes are shown in Online Appendix 3.

## Discussion

This is the first study to use artificial intelligence to identify cardiac rhythm devices from radiographs. This convolutional neural network delivers performance that is at least as good as that of cardiologists using the best available flow chart algorithm. For images which it has never seen, the network identifies the manufacturer of the device with an accuracy of 99.6%, with corresponding cardiologist accuracies ranging from 62.3% to 88.9%. The network can also identify the specific model group with an accuracy of 96.4%.

Just like the flow chart algorithm, whose use it can replace, the neural network has been made publicly and freely available for use.

### Immediate clinical applications

A tool that is faster and at least as reliable as a cardiologist following a flow chart could be useful in several clinical situations. Physicians and physiologists could use it to make a quick assessment of the nature of a cardiac device from a simple chest radiograph. Pacemaker programmers are portable but bulky, and only the manufacturer, the specific programmer, would be able to communicate with the patient’s device. Knowing which programmer to bring saves valuable clinical time. Not only may this facilitate rapid interrogation of a device in an emergency, but also the provision of emergency treatment, such as the delivery of anti-tachycardia pacing in a patient presenting with ventricular tachycardia.

### Human learning from machine learning: Saliency maps

Machine learning has gained a reputation as “black box” technology, which does not provide insights to further human understanding 17, 18. More recently, however, saliency mapping has been developed to provide a useful window into how the neural network is making its decisions. In saliency mapping, the pixels in an image are ranked based on the strength of their effect on the network’s decision (13).

In Figure 3, most humans and, indeed most expert cardiologists, have difficulty in differentiating between the 2 models of pacemaker. However, not only does the neural network accurately distinguish between them but the saliency map highlights the feature that distinguishes them most clearly. Moreover, once this salient feature is pointed out to humans (Figure 4), they now find it straightforward to make the distinction.

Online Appendix 3 shows saliency maps for every model group. Studying these may assist clinicians by using it to sharpen their eye for cardiac device identification.

### Network architecture greatly affects performance

Table 2 shows markedly different levels of performance across different neural network architectures. Of the neural network designs that launched machine learning into prominence, VGGNet is the only 1 still in common use because of its elegant simplicity yet relatively good performance. Surprisingly, however, its performance on this task was poor. This may reflect the necessity for more advanced neural network components, such as “residual connections” and “dimensionality reduction” through “1 × 1 convolutions.”

ResNet was the design that pioneered residual connections, which constitute a method that makes available the original image to all subsequent layers of the network rather than only the first layer. Separately, GoogLeNet Inception was the pioneer for condensing information between layers using 1 × 1 convolutions so that the network’s sophistication was less constrained by the handling of large numbers of parameters.

The design that performed best, however, was Xception, the 1 that made extensive use of both of these innovations, residual connections and 1 × 1 convolutions.

### Study limitations

This neural network recognizes devices in common use in our region of the United Kingdom. However, it will not be able to identify devices not listed in Table 1. For example, the network at present is only trained on 2 implantable loop recorders from 1 manufacturer. However, the network is capable of continuous augmentation. Only 25 examples of a new device are needed to train the neural network.

Therefore, readers are invited to contribute images of other cardiac device types. This can be done conveniently through the Web interface and will be acknowledged on the website when that model of devices is identified for a future website visitor.

This study demonstrates this neural network has superior accuracy in identifying the manufacturer of a device compared with that of human cardiologists and electrophysiologists using a flowchart approach. However, it was found humans performed less well on the present testing dataset than reported previously (3). Reasons for this may include the fact the flowchart algorithm has not been updated in 8 years and the relative abundance of ICDs in the dataset, relative to which it was validated (47.1% vs. 24.4% for this study) and against which the flowchart algorithm appears to perform particularly well (Figure 4 of the original publication).

The accuracy for identifying the model group in the present study is only 96.4%. Furthermore, the “real world” accuracy may differ slightly from this (either better or worse) depending on the distribution of pacemaker model groups in the population. For example, if the neural network performs relatively well on the most popular model groups and relatively poorly on more rarely used devices, the accuracy may be higher than quoted in this paper. For this reason, some studies “weight” their training and testing datasets by the prevalence of classes in the population to give a more accurate representation of real-world performance. Unfortunately, however, no data exist describing the relative incidence of pacemaker models in the population, and so the present dataset must assume balanced class sizes. Fortunately, in clinical practice, the most urgent question is the device manufacturer so that the correct programmer can be brought to the patient’s bedside. There was only 1 classification error for this endpoint across the entire dataset, corresponding to an accuracy of 99.6%, which is less likely to change dramatically with the distribution of devices in the population.

Sometimes, neural networks can come to the wrong conclusion. The present website assists humans by displaying, alongside the medical staff’s uploaded image, not only an image of the model the network thinks this is but also images of the 2 most similar alternatives. These authors have found that, although the network’s selection is correct only 96.4% of the time, in a (coincidental) 99.6% of occasions the correct model group is 1 of those top 3 displayed.

With all neural networks, there is a risk of “overfitting.” This refers to a phenomenon where the neural network becomes excellent at recognizing images it has seen before and been trained on but much less well on real-world examples. It could be explained as the neural network “memorizing” individual images rather than actually “learning” how to tell devices apart. The authors have tried to minimize the risk of overfitting in 2 ways. First, the network’s performance has been defined as its accuracy on the “test set” on which it was not trained. Second, various methods of “regularization” have been included in the network such as dropout and weight decay. These methods penalize the model for making decisions based on only a few very specific elements of an image and, instead, favor training the model to look at a larger variety of features present.

Deployment from “bench to bedside” can be difficult with neural networks, because the large processing power needed is not always present at the point of care. This was mitigated by providing an online Web portal that anyone could use (19).

## Conclusions

This study demonstrates a convolutional neural network is able to accurately identify the manufacturer and model of a cardiac rhythm device from a radiograph. Furthermore, its performance significantly exceeds that of a cardiologist using a flowchart approach.

Perspectives **COMPETENCEY IN MEDICAL KNOWLEDGE:** Machine learning and artificial intelligence are providing rapid developments in medicine, especially in the field of medical image analysis. Our approach may speed up the diagnosis and treatment of patients with cardiac rhythm devices, but this paper also demonstrates how neural networks are increasingly being deployed to process large quantities of medical data throughout the health care system, and how future patient care will likely rely increasingly on computer-aided decision making.

**TRANSLATIONAL OUTLOOK:** Translating achievements in machine learning from ‘bench to bedside’ (or the computing laboratory to the point of care) has often proved difficult. With this study we have provided an educational online portal where physicians can interact with the network online. However, as always, further clinical studies will be essential in assessing the network’s ‘real world’ accuracy before it can be deployed as a validated clinical tool.

## Appendix

Online Appendices 1–3
