# Supplementary material for: Cardiac Rhythm Device Identification Using Neural Networks
Source: JACC Clin Electrophysiol. 2019 May;5(5):576–86. doi: 10.1016/j.jacep.2019.02.003 (PMC6537849; doi:10.1016/j.jacep.2019.02.003)

Appendix 1 – Classes including multiple devices due to identical appearances

## Biotronik - Actros and Philos

Across:


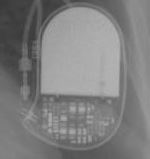


Philos:


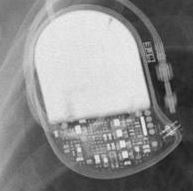


## Boston Scientific - Altrua and Insignia

Altrua:


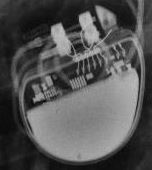


Insignia:


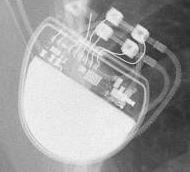


## Boston Scientific - Autogen, Cognis, Energen and Teligen,

Autogen:


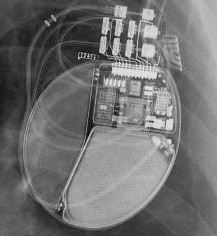


Cognis:


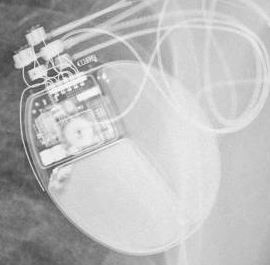


Energen:


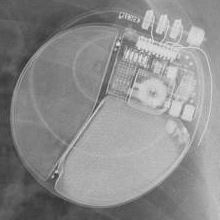


Teligen:


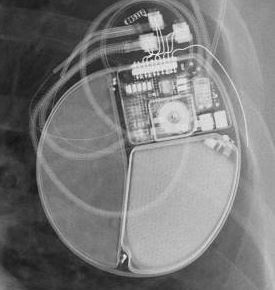


## Boston Scientific – Contak TR, Discovery, Meridian and Pulsar Max

Contak TR:


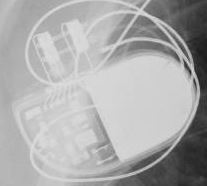


Discovery:


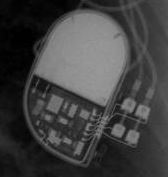


Meridian:


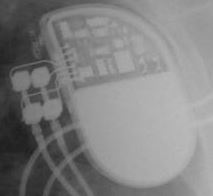


Pulsar Max:


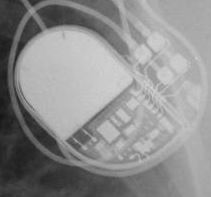


## Medtronic – Adapta, Kappa, Sensia and Versa

Adapta:


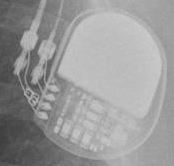


Kappa:


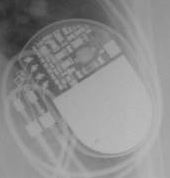


Sensia:


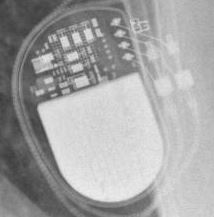


Versa:


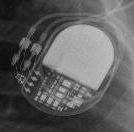


## Medtronic - C20 and T2O

C20:


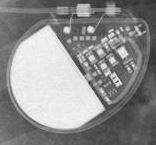


T20:


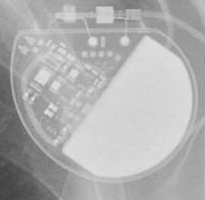


## Medtronic – Claria, Evera, Viva

Claria:


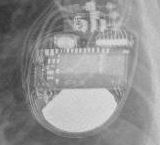


Evera:


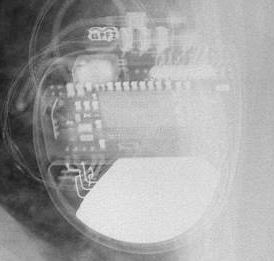


Viva:


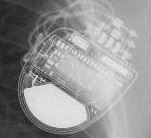


## Medtronic – Concerto, Consulta, Maximo, Protecta, Secura

Concerto:


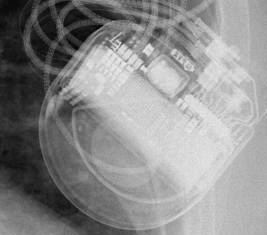


Consulta:


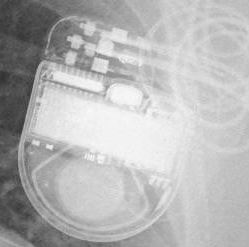


Maximo:


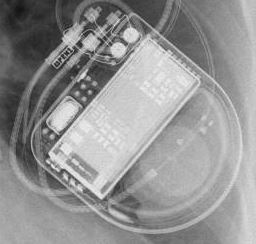


Protecta:


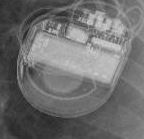


Secura:


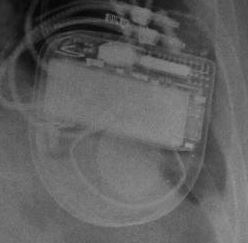


## Sorin Rhapsody and Symphony

Rhapsody:


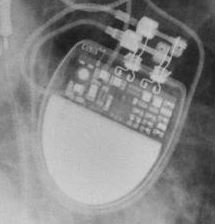


Symphony:


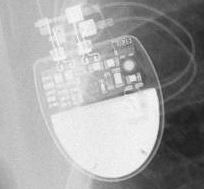


## St. Jude – Quadra Assura and Unify

Quadra Assura:


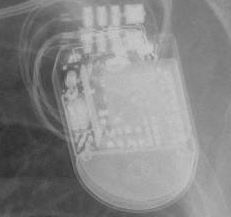


Unify:


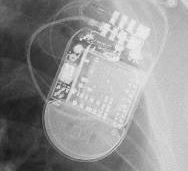


Appendix 2 – Incorrectly-predicted examples from the test set (n=8)

#
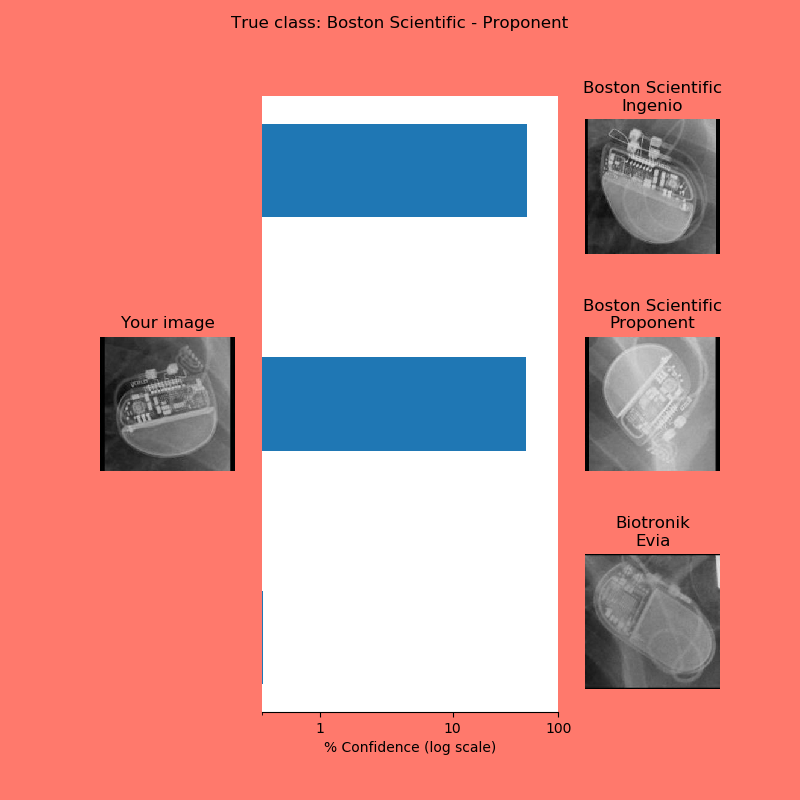

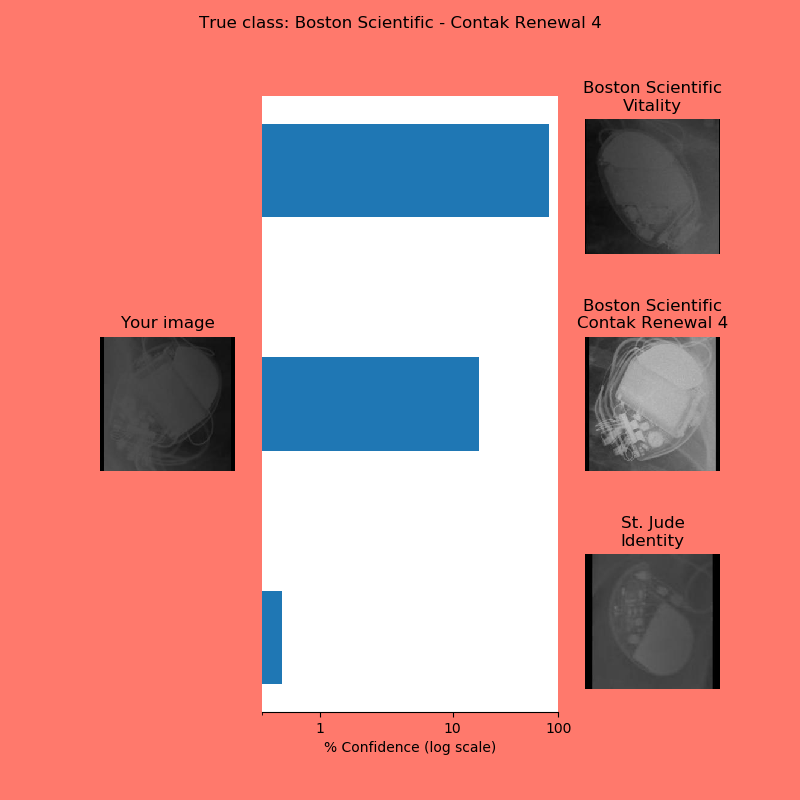


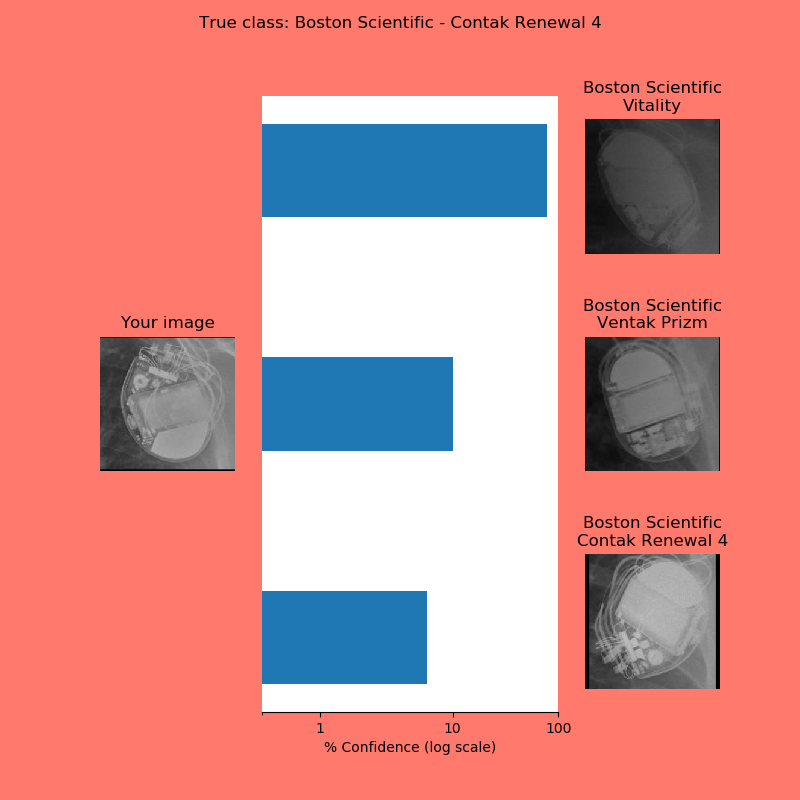


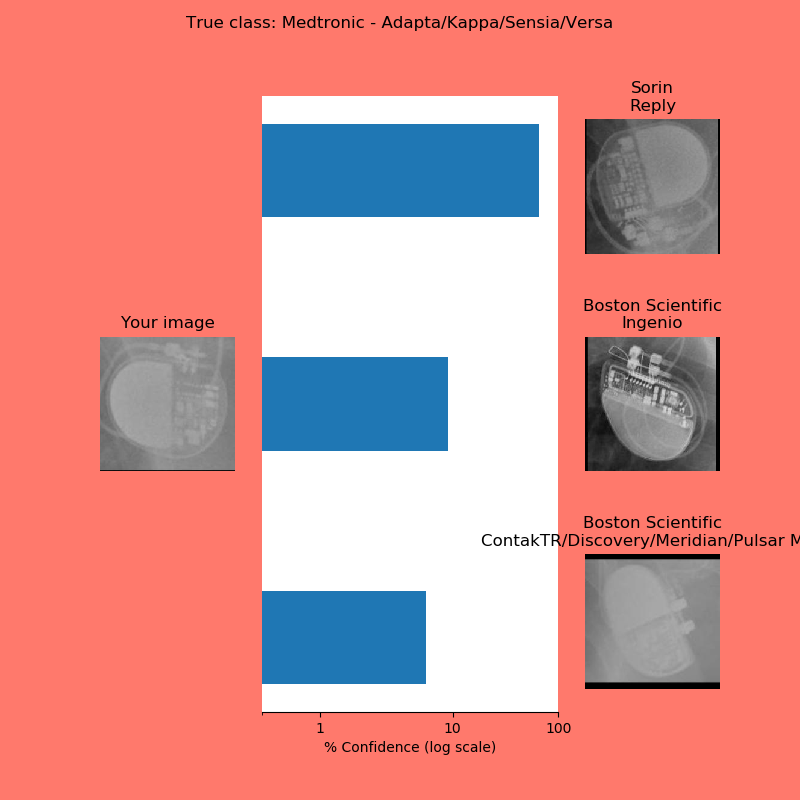

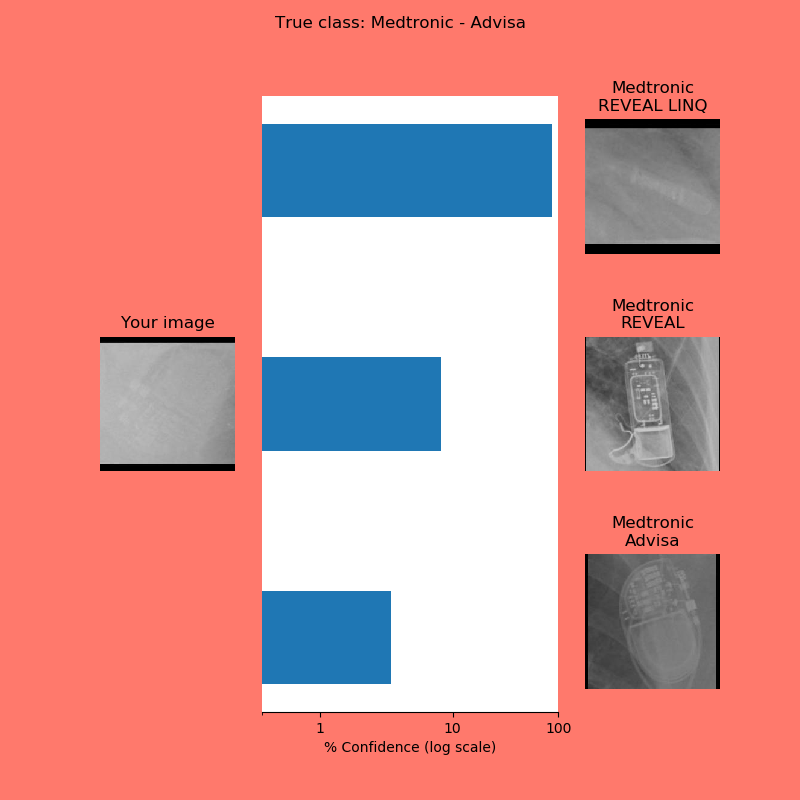

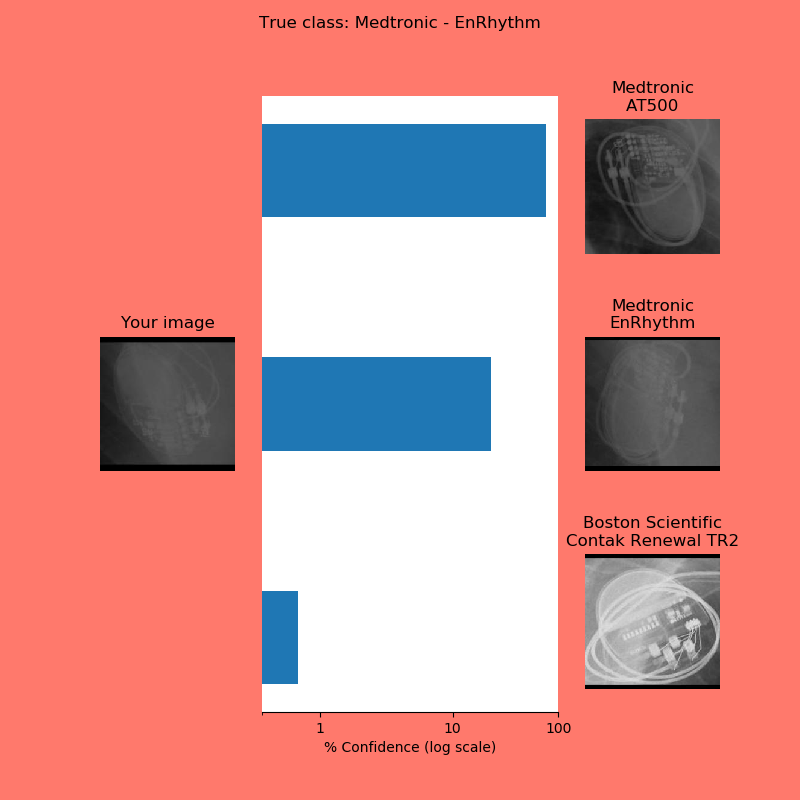

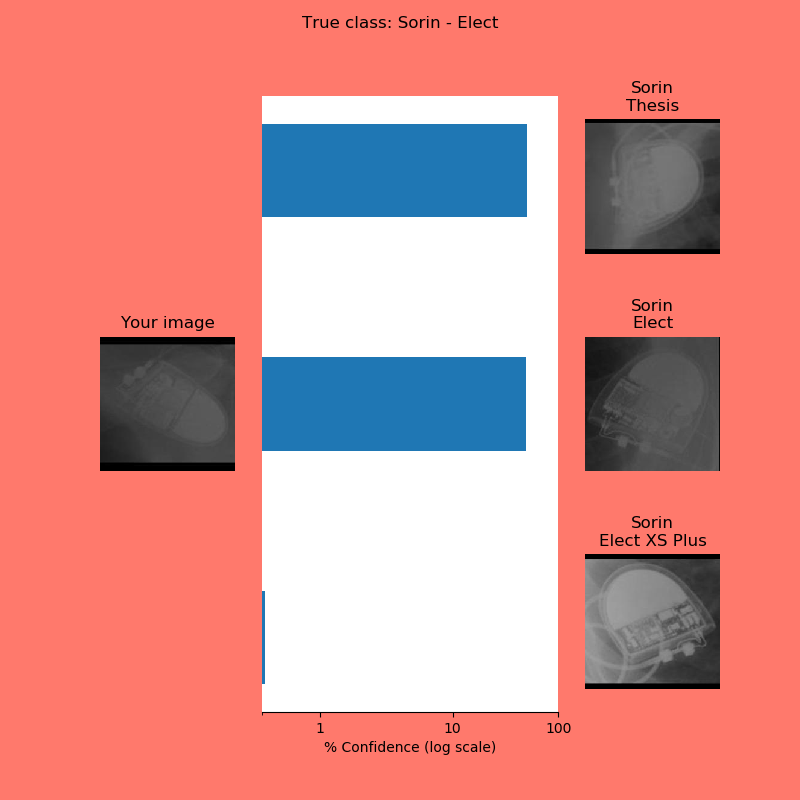


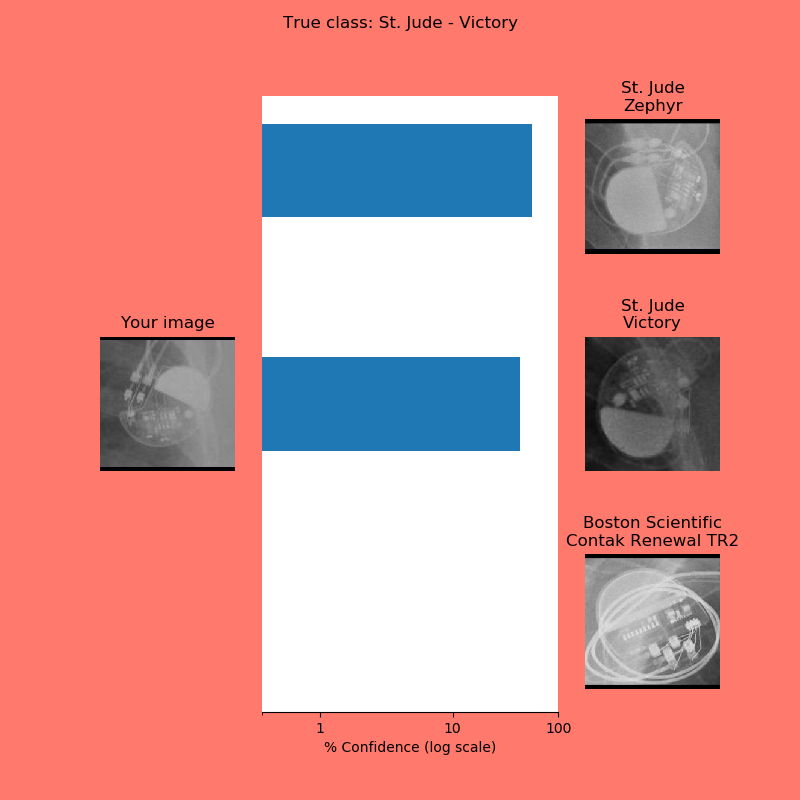


Appendix 3 – Representative saliency maps from the test set


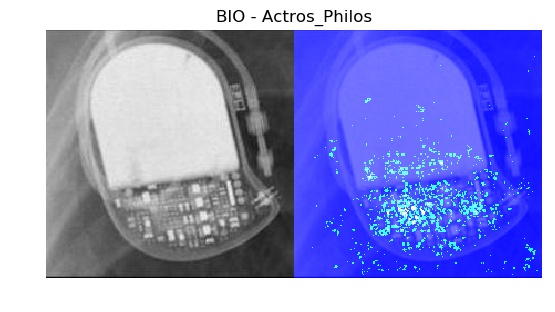


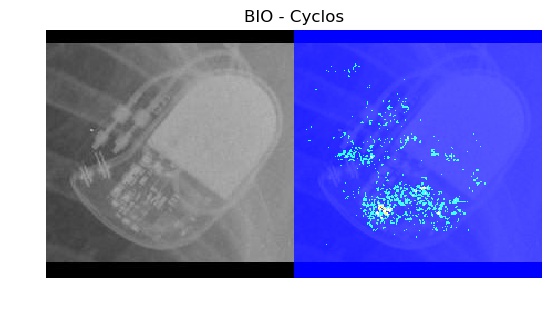


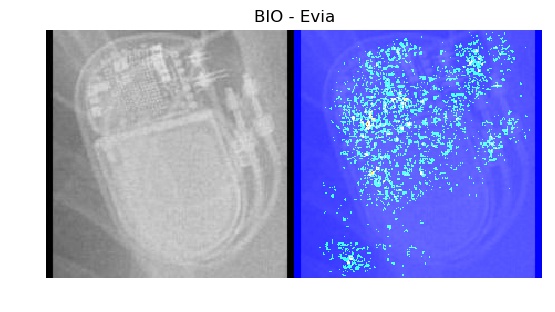


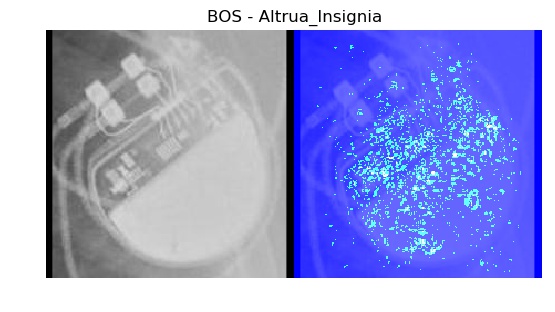


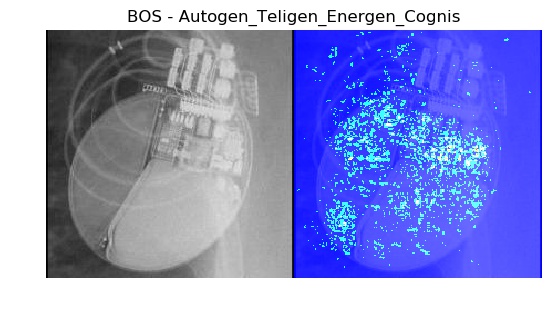


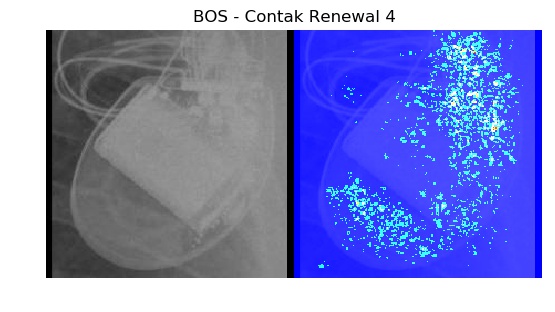


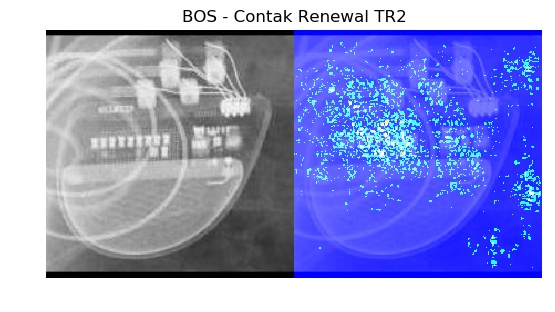


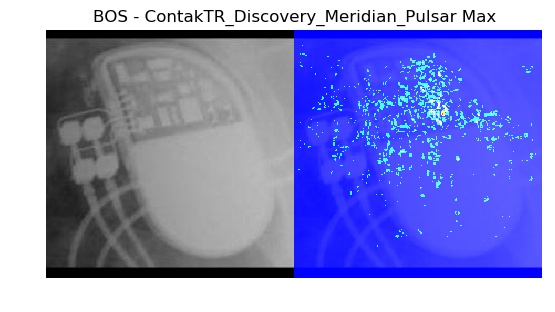


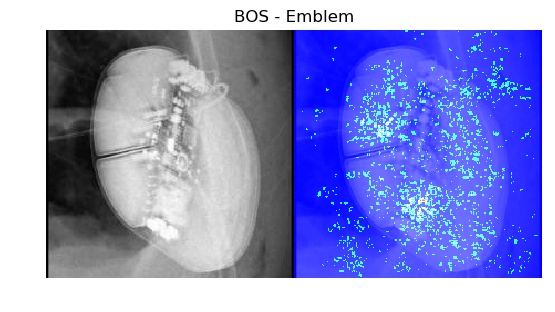


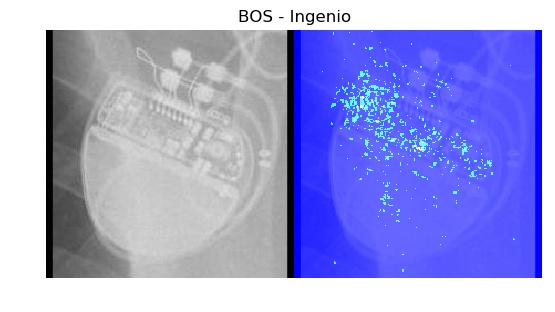


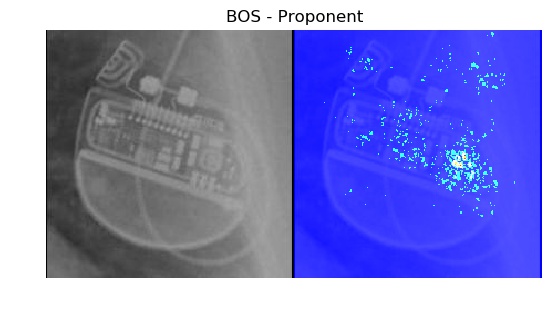


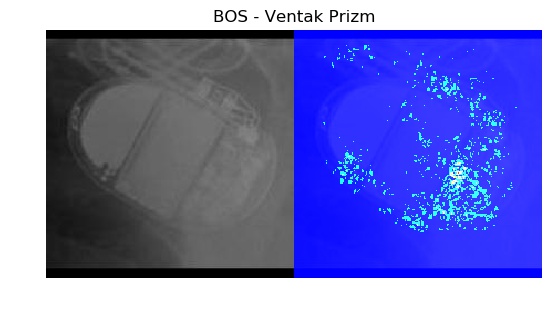


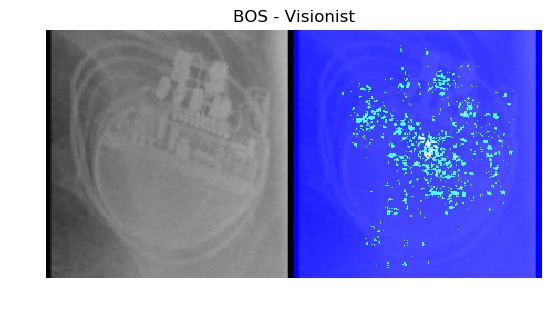


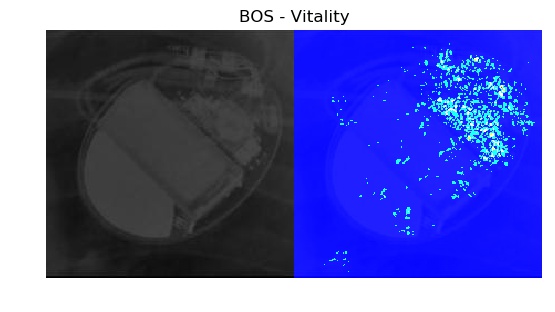


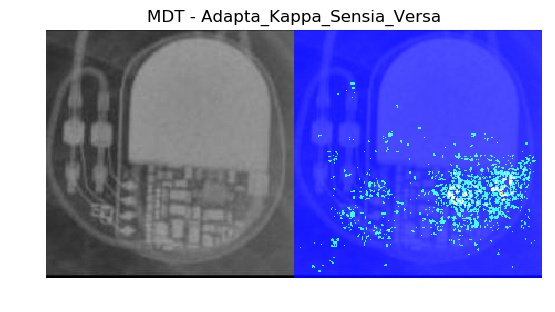


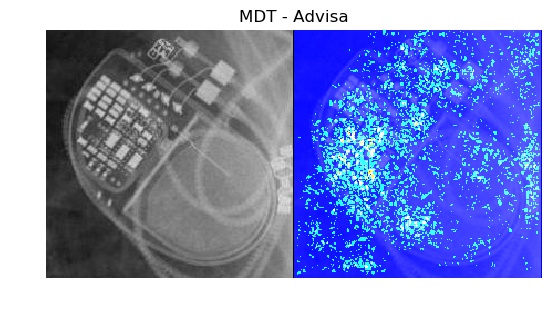


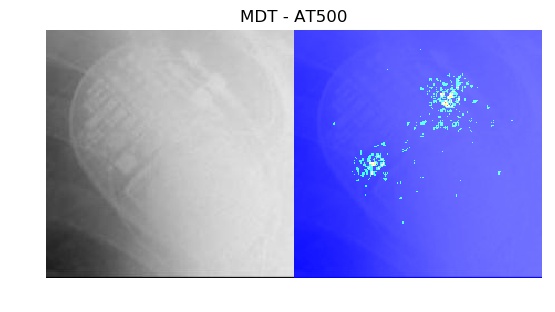


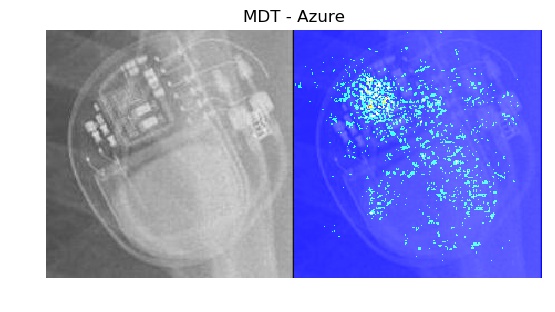


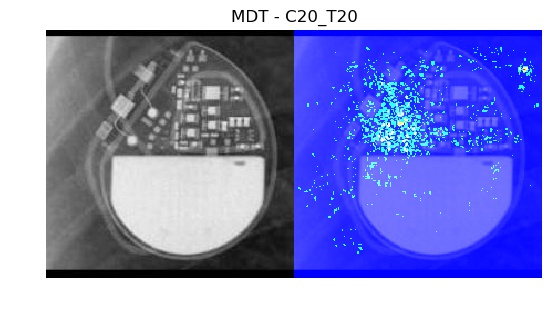


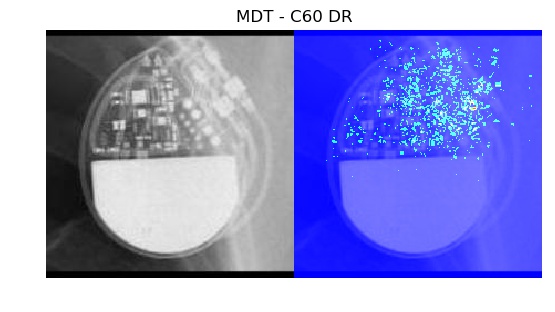


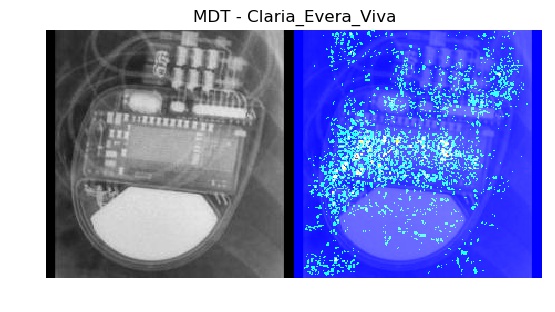


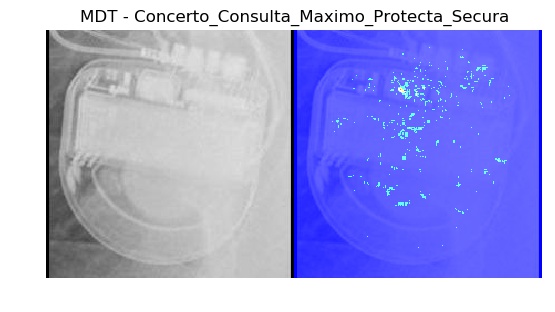


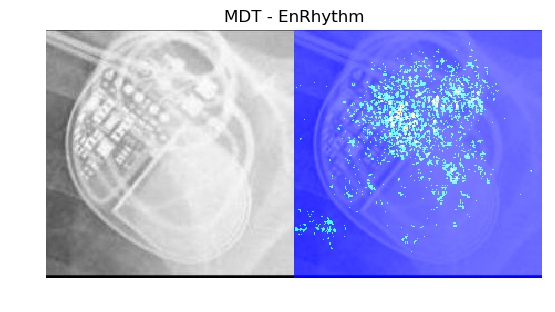


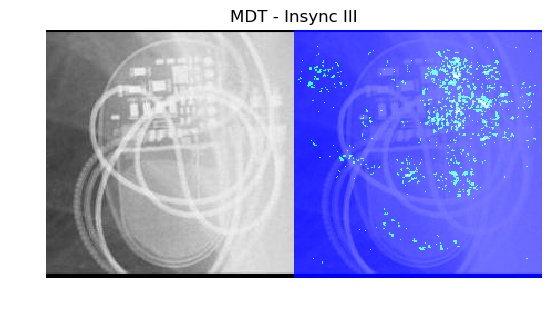


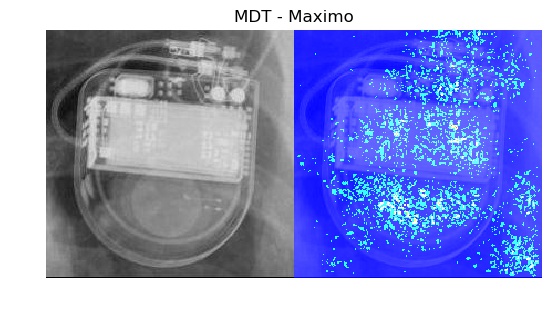


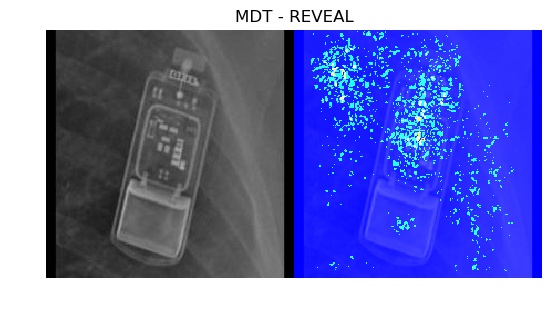


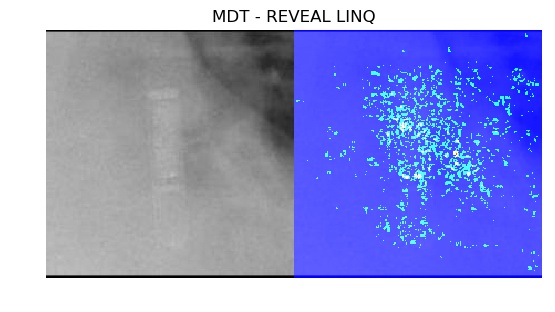


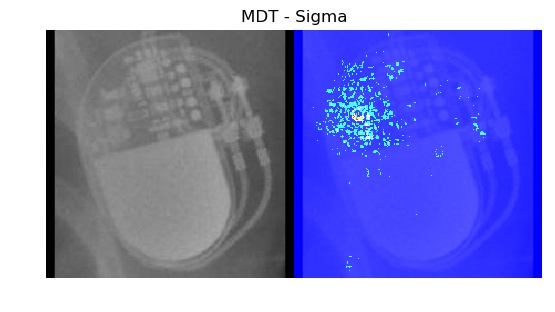


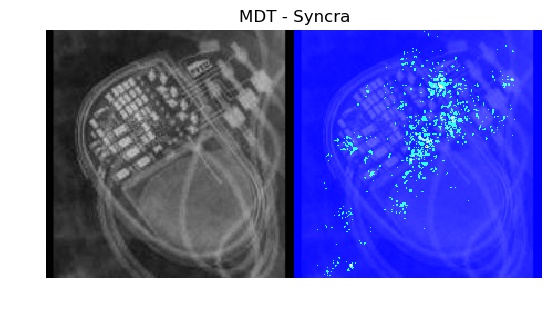


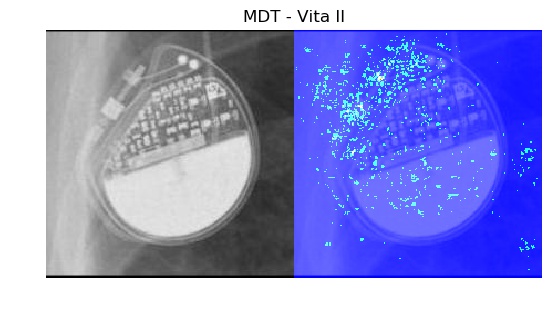


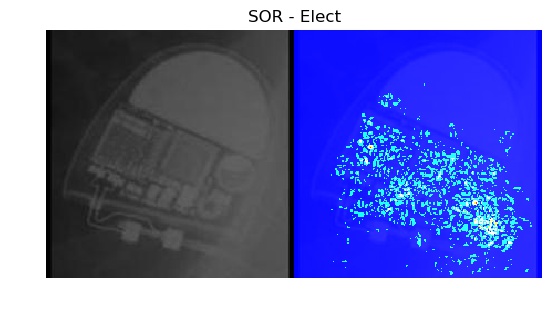


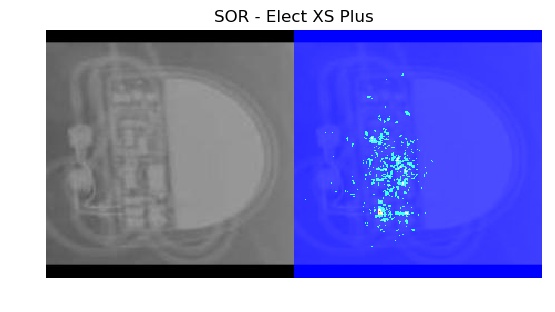


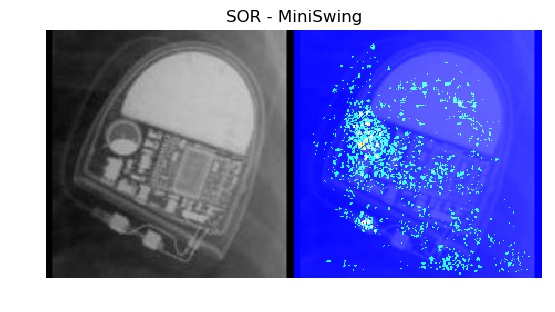


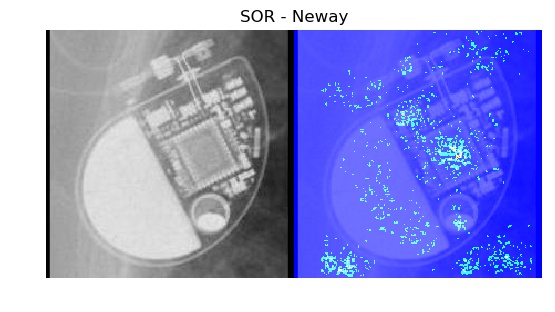


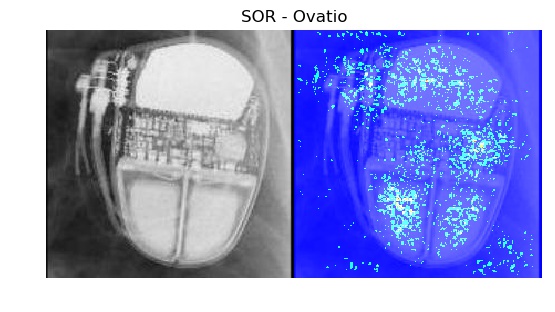


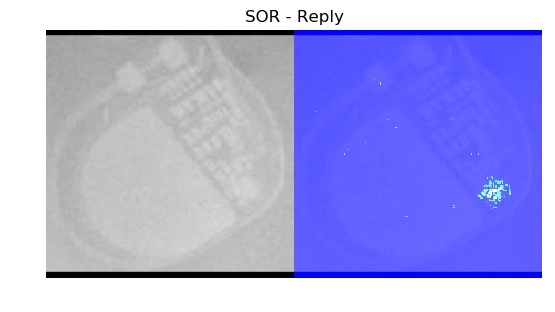


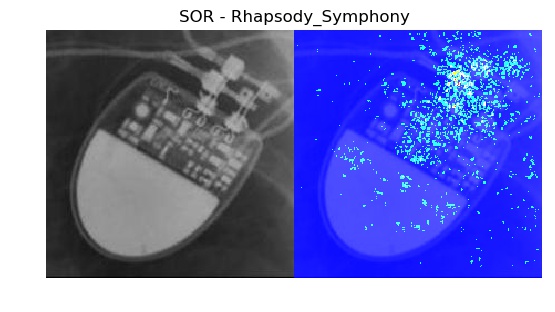


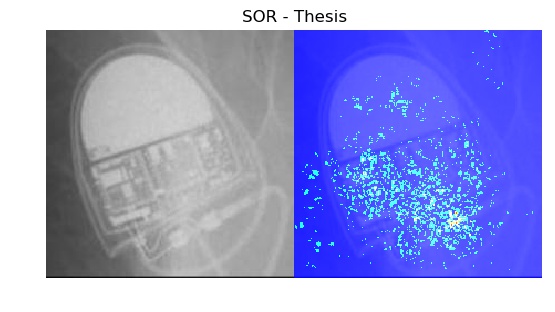


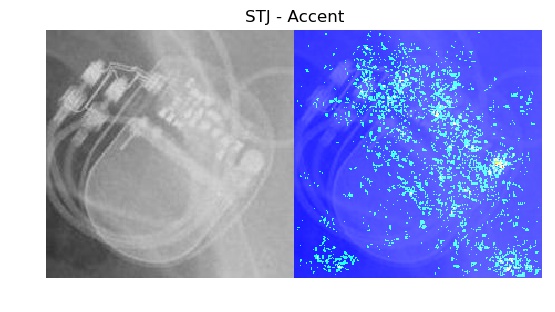


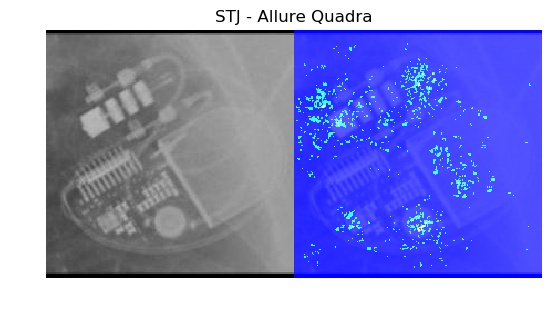


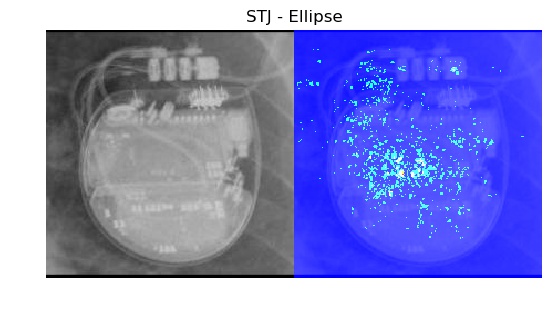


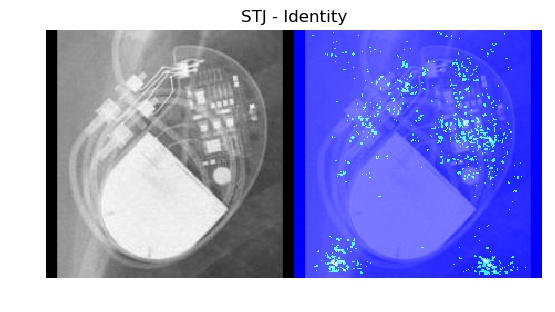


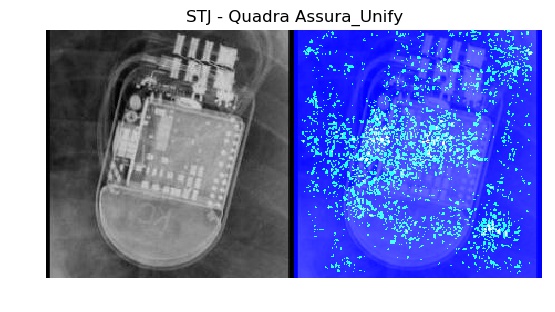


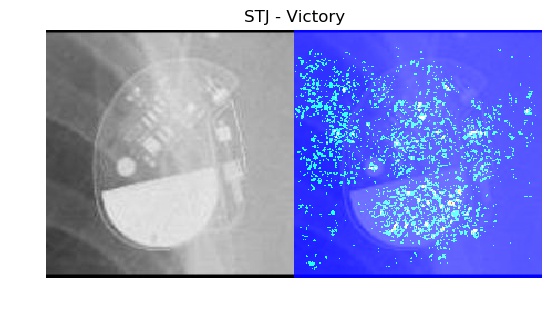


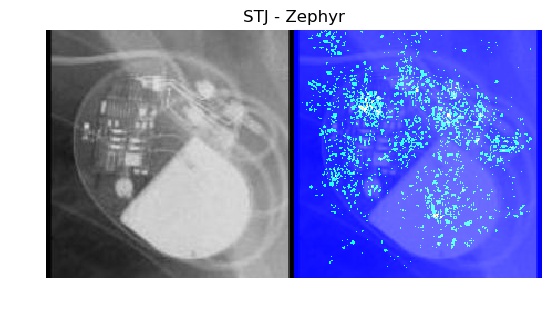

Supplement: Online Appendices 1–3 [file mmc1.docx]
